# The Role of Nitric Oxide in Plant Responses to Salt Stress

**DOI:** 10.3390/ijms23116167

**Published:** 2022-05-31

**Authors:** Jian-Xiu Shang, Xiaoying Li, Chuanling Li, Liqun Zhao

**Affiliations:** School of Life Sciences, Hebei Normal University, Shijiazhuang 050024, China; shangjianxiu@hebtu.edu.cn (J.-X.S.); xying0506@163.com (X.L.); chuanlingzz@163.com (C.L.)

**Keywords:** plant, nitric oxide, salt tolerance

## Abstract

The gas nitric oxide (NO) plays an important role in several biological processes in plants, including growth, development, and biotic/abiotic stress responses. Salinity has received increasing attention from scientists as an abiotic stressor that can seriously harm plant growth and crop yields. Under saline conditions, plants produce NO, which can alleviate salt-induced damage. Here, we summarize NO synthesis during salt stress and describe how NO is involved in alleviating salt stress effects through different strategies, including interactions with various other signaling molecules and plant hormones. Finally, future directions for research on the role of NO in plant salt tolerance are discussed. This summary will serve as a reference for researchers studying NO in plants.

## 1. Nitric Oxide (NO) Biosynthesis under Saline Conditions

In plants, NO is produced mainly through two enzymatic pathways: oxidative and reductive. In the oxidative pathway, L-arginine (with oxygen and NADPH) is converted to NO and citrulline via the action of NO synthase (NOS); however, the actual existence and identity of NOS in plants are currently unresolved. *Arabidopsis* NO-associated 1 (AtNOA1) was originally reported to possess NOS activity; however, subsequent studies found this to be incorrect [1]. NOS-like activity has been reported in plant mitochondria and peroxisomes, and animal NOS inhibitors can significantly inhibit NO synthesis in plants, suggesting the existence of a NOS-like pathway for NO production in plants [2]. In the reductive pathway, NO is generated by nitrate reductase (NR) through the successive reduction of nitrate to nitrite and then to NO with NADH as an electron donor. *nia1/2* double-mutant plants exhibit a reduced endogenous NO level, indicating that *NIA1/2* are the major NR genes related to NO production in plants [3].

On the other hand, cells utilize various mechanisms to remove NO. For example, NO reacts with glutathione (GSH) to form *S*-nitrosylated GSH (GSNO), a major bioactive NO species. GSNO is then reversibly metabolized to oxidized GSH (GSSG) and ammonium (NH_4_^+^) by a highly conserved *S*-nitrosoglutathione reductase (GSNOR); thus, the NO level is dynamically modulated in plants [4].

NO, as a major signaling molecule, plays an important role in plant growth and development and in plant resistance to various environmental stimuli, including salt stress [5]. Under saline conditions, plants promote NO synthesis mainly by increasing the activity of a NOS-like enzyme or NR and by inhibiting the activity of GSNOR.

As early as 2004, Zhao et al. [6] reported that sodium chloride (NaCl) treatment increased NOS activity and NO release in dune reed calli. The inhibition of NO production using specific inhibitors further indicated that NO is produced via NOS activity under saline conditions. Later, increased NOS activity and NO levels were demonstrated in salt-stressed rice and maize [7,8]. Overexpression of rat neuronal NOS (nNOS) in rice increased both NOS activity and NO accumulation in transgenic plants and improved the tolerance of the plants to salt stress [7]. Recently, it was also reported that an organic plant growth-promoting substance, 5-aminolevulinic acid, triggered NO synthesis in maize through NOS activation, resulting in improved salt tolerance [8]. Together, these data indicate that NOS-produced NO plays a positive role in the resistance of plants to saline conditions.

However, exposure to NaCl was also shown to reduce the quantity of NOA1 in *Arabidopsis*, leading to a decrease in endogenous NO levels measured using NO-specific fluorescent probes; this result might be plant type- or measurement method-specific [9].

Overall, these studies suggest that NOS-like activity plays an essential role in NO production under conditions of salt stress; still, the modulation of NR activity under high-salt conditions has also been confirmed in plants. Indeed, Reda et al. [10] proved that increased NO production under salt stress depends on NR. Salt increases NR activity at the transcriptional and post-transcriptional levels to promote NO production in cucumber roots, and it induces glucose-6-phosphate dehydrogenase (G-6-PDH) protein expression and enzyme activity, as well as NR activity and NO production, in red kidney bean roots. By using specific inhibitors, it has been shown that G-6-PDH plays a pivotal role in NR-dependent NO production, and it establishes root tolerance to salt stress [11].

The above data are consistent with reports showing that most NO production is attributable to NOA1/NR [12]; however, salt-stressed plants not only promote NO production by activating a NOS-like enzyme or NR, but they also inhibit NO degradation so as to promote NO accumulation. Zhou et al. [13] previously reported that the level of NO varied in response to salt treatment depending on calmodulin 1/4 (AtCaM1/4) expression. AtCaM1 and AtCaM4 promote plant salt resistance via increased NO accumulation by binding to and inhibiting GSNOR following salt exposure [13]. Further, Sun et al. [14] showed that the *Arabidopsis* membrane trafficking-related protein patellin1, which forms a complex with CaM4 and plays a regulatory role in plant freezing responses, positively modulates NO accumulation in the presence of salt.

In summary, plants regulate NO production under salt stress via multiple pathways, which may be a result of long-term adaptation to the environment. However, there is still a huge blind spot in understanding the processes and pathways of NO synthesis in plants under salt stress; it is one of the most challenging issues in the field of NO research.

## 2. NO Alleviates Salt Stress-Induced Damage

**Figure 1:**
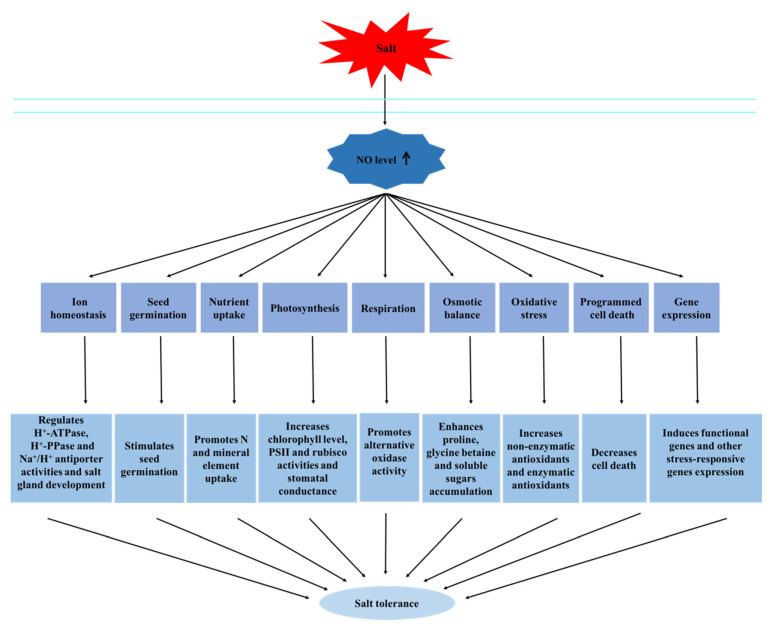
NO mediates salt tolerance through different strategies.

Salt stress decreases the height, fresh weight, total dry weight, photosynthetic pigment content, and protein content of plants. The inhibitory effects of salt stress can be alleviated by exogenous NO treatment in wild barley [15]. Exogenous application of SNP increased the fresh weight and shoot/root elongation of *Nitraria tangutorum* seedlings under salt stress. Leaf senescence and root damage induced by salt stress were also alleviated. Meanwhile, application of the NO scavenger cPTIO and mammalian NOS inhibitor L-NAME significantly worsened stress-induced damage under high-salt conditions [16]. In plants, NO alleviates the damage caused by salt exposure via several strategies (Figure 1), as summarized below.

### 2.1. Ion Homeostasis

Salt stress can cause excessive sodium ion (Na^+^) accumulation and potassium ion (K^+^) pool depletion in plants, resulting in disrupted intracellular ion homeostasis and ion toxicity [17]. Maintaining an optimal Na^+^/K^+^ ratio is crucial to preventing ion toxicity and preserving cytosolic enzyme activity, intracellular osmotic pressure, and membrane potential [18,19]. NO plays an essential role in maintaining ion homeostasis in plants under saline conditions. It helps plants excrete Na^+^ or sequester Na^+^ into vacuoles by regulating the activity of ion channels. In some halophytes, NO promotes the development of salt glands that excrete Na^+^ from the cell.

Salt-stressed plants, including *Kandelia obovata*, pak choi, wheat, rice, and soybean, treated with the NO donor sodium nitroprusside (SNP), show increased endogenous NO levels and selective transport of K^+^ and Na^+^ to maintain K^+^/Na^+^ homeostasis [20,21,22,23,24]. The *Atnoa1* mutant, which exhibits a low NO content, displays a lower K^+^/Na^+^ ratio in its shoot compared to wild-type plants following exposure to NaCl [9]. These data indicate that NO plays a positive role in K^+^/Na^+^ homeostasis under saline conditions.

NO reportedly enhances the hydrolytic activity of plasma membrane (PM)-localized proton pump (H^+^-ATPase), which provides the driving force for improving ion imbalances in salt-stressed wheat plants [25]. The NO-induced gene expression and protein accumulation of PM H^+^-ATPase has been identified in several species, including reed, *N. tangutorum*, *Avicennia marina*, and *K. obovata* [6,16,26,27]. In addition, NO promotes the expression of Na^+^ efflux pumps, such as the PM Na^+^/H^+^ antiporter, to push Na^+^ from the cytoplasm to the extracellular environment; this reduces the Na^+^ content and the Na^+^/K^+^ ratio in *N. tangutorum* and *A. marina* under saline conditions [16,26].

NO stimulates the activity of vacuolar H^+^-ATPase and H^+^-PPase, which provide the driving force for Na^+^/H^+^ exchange under salt stress in wheat leaves and maize seedlings [25,28]. In addition, NO promotes the gene expression and protein accumulation of vacuolar ion transporters, such as the Na^+^/H^+^ antiporter, which sequester cytoplasmic Na^+^ in vesicles so as to reduce the Na^+^ content and Na^+^/K^+^ ratio [16,26].

In addition to regulating H^+^-ATPase, H^+^-PPase, and Na^+^/H^+^ antiporter, NO regulates salt gland development in some halophytes. Researchers demonstrated that NO increased the total number of salt glands that developed from dermatogen cells on adaxial surfaces and the Na^+^ secretion rate per leaf in NaCl-treated *Limonium bicolor* seedlings [29].

In summary, NO stimulates Na^+^ excretion and promotes K^+^ uptake to maintain the cytosolic K^+^/Na^+^ balance by regulating the activity of several enzymes (e.g., H^+^-ATPase, H^+^-PPase, and Na^+^/H^+^ ion exchanger) or promoting salt gland development, but how NO regulates these processes requires further investigation.

### 2.2. Seed Germination

Seed germination is the first step in the life cycle of angiosperms. Salt inhibits seed germination, thereby causing great damage to agricultural production. Several reports have shown that the application of SNP under saline conditions increases the percent germination of various plant species, including *Eucalyptus urophylla*, wheat, and rice [30,31,32]. Another study found that NO alleviated the decreases in germination percentage, germination index, vigor index, and imbibition rate of wheat seeds exposed to salt stress, mainly by increasing beta-amylase activity [31]. Low concentrations of NO prevent salt-inhibited seed germination, while high concentrations of NO exacerbate salt-inhibited seed germination in rice [32]. These findings indicate that the exogenous application of NO to prevent salt-inhibited germination requires a suitable concentration.

### 2.3. Nutrient Absorption

Salt affects the uptake of nutrients by plants, whereas external NO application promotes the uptake of beneficial nutrients by plants. For example, the application of NO increased the nitrogen (N) content in rice leaves and alleviated the inhibitory effects of salt stress on plant height and biomass accumulation [33]. NO also regulates the expression of NH_4_^+^ transporters to mediate NH_4_^+^ transport, which may reflect N uptake and its subsequent utilization [33]. Another report showed that mineral element (i.e., Zn, Fe, B, K, Ca, and Mg) uptake was increased by NO treatment in *Capsicum annum* under saline conditions [34]. These data indicate that NO might have a beneficial impact on the growth of plants under salinity stress by regulating mineral nutrient intake.

### 2.4. Photosynthetic Efficiency

Salt stress severely impacts photosynthesis by decreasing chloroplast activity, the photosynthetic rate, and stomatal conductance [35,36]; however NO has been shown to reverse many of the adverse effects of salt on the photosynthetic machinery of plants. For instance, NO enhanced the photosynthetic parameters and machinery in salt-stressed tomato plants [37]; moreover, the effects of salt stress on photosynthetic performance were mitigated more efficiently when NO was applied together with the split application of N and sulfur (S) [38]. Meanwhile, Sami et al. [39] noted that the application of SNP to mustard plants improved several photosynthetic attributes, including the chlorophyll level, chlorophyll fluorescence, and gas exchange parameters. In salinized eggplant, a decrease in photosystem II (PSII) activity was associated with the inactivation and destruction of the PSII reaction center. However, exogenous NO alleviated this effect [40]. Similarly, NO application increased the chlorophyll content in salt-stressed pea, bermudagrass, and soybean plants [41,42,43]. Moreover, although the chloroplast thylakoid system is distorted in salinized plants, combined SNP and S supplementation positively affected the chloroplast structure in salinized mustard plants, restoring them to a normal shape, and the thylakoid system was appropriately rearranged [44].

NO also increases Rubisco activity. For example, NO protected the photosynthetic capacity of Indian mustard against salt stress [45]. A proteomic study found that under high-salt conditions, the abundance of photosynthesis-related proteins, including ribulose-phosphate 3-epimerase, Rubisco large subunit (RBCL), Rubisco activase A, and quinine oxidoreductase-like protein isoform 1 (QOR1), was significantly decreased; however, the abundance of proteins such as RBCL and QOR1 was increased by SNP [46].

In addition, salt exposure causes stomatal closure, which decreases the availability of carbon dioxide (CO_2_) in leaves and reduces carbon fixation. The application of exogenous NO to salt-stressed plants improved such photosynthetic parameters as the intrinsic CO_2_ concentration, stomatal conductance, transpiration, and photosynthetic rate [34,47].

In brief, NO improves the photosynthetic parameters and machinery, including the chlorophyll content, PSII activity, Rubisco activity, and stomatal conductance in salt-stressed plants to alleviate salt damage.

### 2.5. Respiration

Alternative oxidase (AOX), a unique respiratory terminal oxidase in plants, influences salt-stress responses by catalyzing cyanide-resistant respiration. NO enhances both the expression of *AOX* genes and the cyanide-resistant respiration rate, which are induced by salt stress, to alleviate the oxidative and photosynthetic damage caused by salt exposure [48].

### 2.6. Osmotic Balance

High environmental levels of salt produce a low water potential, resulting in osmotic stress in plants. To maintain cell volume and vigor, plants accumulate osmotic substances in the cytoplasm (mainly low-molecular-weight compounds, including charged metabolites [e.g., proline and glycine betaine], polyols [e.g., mannitol and sorbitol], simple sugars [e.g., sucrose and fructose], and complex sugars [e.g., alginate and cottonseed]) for osmoregulation [49].

In response to salt treatment, NO can increase the contents of proline, glycine betaine, and soluble sugars in plants, thereby alleviating osmotic stress caused by salt exposure.

Evidence indicates that NO raises the proline level to maintain the osmotic balance in *K. obovata*, rice, *Kosteletzkya virginica*, *Solanum lycopersicum*, and *Brassica juncea* [20,33,50,51,52]. NO also increases the contents of proline and soluble sugars in pak choi and tomato [21,53]. In rice, NO was shown to significantly increase the activities of sucrose synthase and sucrose phosphate synthase under saline conditions, resulting in sucrose accumulation and increased salt tolerance [33]. NO promotes proline and glycine betaine accumulation in mustard [54], and it can mitigate the harmful effects of high-salt conditions on chickpea plants by improving the levels of osmolytes (proline, glycine betaine, soluble proteins, and soluble sugars) [55].

### 2.7. Oxidative Stress

Salt stress causes the rapid accumulation of reactive oxygen species (ROS), including superoxide, hydrogen peroxide (H_2_O_2_), hydroxyl radicals, and singlet oxygen. At low concentrations, ROS act as signaling molecules to induce salt stress responses; however, in excess, ROS can cause oxidative damage.

High intracellular concentrations of ROS can negatively impact various physiological processes (e.g., causing protein denaturation, lipid peroxidation, DNA damage, and abnormal carbohydrate accumulation). Such cellular damage can lead to plant growth inhibition. Therefore, plant cells have evolved complex enzymatic and non-enzymatic antioxidant defense mechanisms to scavenge excess ROS in order to control the intracellular ROS concentration and maintain a normal redox state [56,57].

Non-enzymatic scavengers include ascorbic acid (ASA), GSH, alkaloids, alpha-tocopherol, carotenoids, phenolics, flavonoids, and proline. Enzymatic scavengers include superoxide dismutase (SOD), ascorbate peroxidase (APX), GSH reductase (GR), catalase (CAT), peroxidase, and guaiacol peroxidase (GPX) [57].

NO is considered a functional molecule in plants that alleviates salt-induced damage by modulating antioxidant metabolic pathways.

#### 2.7.1. Lipid Peroxidation

Salt stress leads to high H_2_O_2_ levels and lipid peroxidation. Malondialdehyde (MDA), a product of oxidative lipid modification, has a negative impact on membrane properties and functions, including fluidity, protein cross-linking, ion transport, and enzyme activity [5].

NO is a powerful inhibitor of ROS, which can cause lipid peroxidation. SNP application has been shown to decrease the contents of H_2_O_2_, superoxide anion, and MDA in NaCl-stressed pak choi, pepper, mustard, pea, eggplant, tomato, spinach, and barley plants, thereby preventing oxidative damage [21,34,39,40,41,53,58,59].

#### 2.7.2. Antioxidant Defenses

To prevent damage caused by ROS, plants have developed non-enzymatic and enzymatic antioxidant defense mechanisms that increase salt tolerance [60].

**Non-enzymatic antioxidants**

SNP activates the ascorbate (AsA)-GSH cycle in *N. tangutorum* seedlings; the resulting increase in antioxidants helps alleviate oxidative damage caused by salt stress through ROS scavenging [16]. Meanwhile, the reduced AsA/dehydroascorbate (DHAsA) and GSH/GSSG ratios seen in salt-stressed soybean root nodules can be reversed by the application of a NO donor, which increases the levels of reduced antioxidant metabolites [61]. Further, NO increased the contents of reduced ASA, GSH, and polyphenols in tomato, *Aegiceras corniculatum*, and cotton under salt stress [53,62,63] and significantly elevated the levels of several antioxidation-associated compounds, including proline, AsA, GSH, phenolics, and flavonoids, as well as the total antioxidant capacity (indicated by DPPH scavenging activity), in NaCl-treated spinach plants [58].

**Enzymatic antioxidants**

NO protects cells from oxidative damage by increasing the activity of antioxidant enzymes. For example, the application of a NO donor to NaCl-treated plants increased the activity of SOD, CAT, and APX [21,55,59,64]. Application of a NO donor to salt-treated *A. corniculatum* also enhanced the activity of GPX, which catalyzes the detoxification of peroxides and hydroperoxides [62]. SNP treatment increased GR activity in the leaves of salt-treated chickpea and tomato seedlings and the cotyledons of salt-treated sunflower seedlings [55,65,66]. Further, the application of NO increased dehydroascorbate reductase activity and monodehydroascorbate reductase activity, resulting in increased AsA/DHAsA and GSH/GSH disulfide ratios in salinized *Vigna angularis* [67].

NO has a positive effect on the transcript levels of genes encoding antioxidant enzymes. The expression levels of *SOD*, *CAT*, and *APX* increased after treating chickpea plants with NaCl and the NO donor S-nitroso-N-acetyl penicillamine [55].

NO mitigates plant damage through non-enzymatic antioxidants and enzymatic antioxidants to lower the accumulation of ROS and alleviate lipid peroxidation under salt stress.

### 2.8. Programmed Cell Death (PCD)

PCD maintains cellular homeostasis by eliminating old, damaged, or unwanted cells. In plants, PCD takes place during development and in response to biotic and abiotic stresses [68].

It has been reported that NO decreases PCD in salt-treated mustard plants [39]. However, detailed studies of NO, PCD, and salt tolerance are scarce; more work is needed.

### 2.9. Gene Expression

In addition to promoting the expression of functional genes such as those encoding H^+^-ATPase, SOD, CAT, and APX [16,28], NO regulates the expression of other stress-responsive genes to alleviate the negative effects of salt stress on plants. For example, NO regulates the expression of sucrose transporters; this may provide energy and structural substances to support the growth and development of young leaves [33]. In nNOS-overexpressing plants, the expression of several stress-responsive genes, including *OsDREB2A*, *OsDREB2B*, *OsSNAC1*, *OsSNAC2*, *OsLEA3*, and *OsRD29A*, was found to be increased under high-salt conditions [7]. These results, taken together, suggest that both the external application and internal production of NO can regulate the expression of stress-responsive genes under stressful conditions, thereby promoting increased salt tolerance. To date, however, reports on the NO-mediated regulation of gene expression in salt-stressed plants are limited; further research is required.

## 3. NO Signaling under Conditions of Salt Stress

Many small active substances are essential for plant growth and development, including polyamines (PAs), melatonin, carbon monoxide (CO), calcium ions (Ca^2+^), and S. These substances and NO play important roles in integrating salt signaling by regulating various biochemical processes. In addition, phytohormones have been shown to work together with NO to affect salt tolerance by modulating several physiological processes and biochemical mechanisms, including photosynthesis, mineral nutrient homeostasis, osmolyte accumulation, and antioxidant metabolism.

### 3.1. NO and Small-Molecule Active Substances

#### 3.1.1. PAs

PAs are aliphatic polyatomic molecules distributed ubiquitously in plants. Several studies have shown that crosstalk between PAs and NO improves salt tolerance in plants [69,70].

Tailor et al. [69] reported that NO upregulates PA biosynthetic enzymes, including arginine decarboxylase and S-adenosylmethionine decarboxylase, under salt stress. Fan et al. [70] found that NO also enhances the salt tolerance of cucumber seedlings by regulating the free PA content. Additionally, NO reduces the activity of PA oxidase to inhibit PA degradation under high-salt conditions in sunflower cotyledons [69]. Therefore, NO positively regulates PA homeostasis to help plants resist salt stress. However, the mechanisms underlying plant tolerance to salt stress through the association of NO and PA require further study.

#### 3.1.2. Melatonin

Melatonin is a small molecule that acts as a free radical scavenger and antioxidant under stressful conditions, and many studies have shown that it is closely related to NO in the salt responses of plants. Zhao et al. [71] found that the exogenous application of melatonin and SNP reduced Na^+^ accumulation to alleviate the salt-induced inhibition of rapeseed growth; however, melatonin could not rescue the salt hypersensitivity of *Atnia1/2* and *Atnoa1* mutant plants [71]. Subsequent experiments revealed that cPTIO prevented the melatonin-induced expression of antioxidant genes, including *APX* and *SOD*, in NaCl-stressed rapeseed root tissues [71]. These data indicate that NO may act downstream of melatonin to promote salt tolerance.

However, the interplay between melatonin and NO in the response of plants to salt stress is controversial. For example, multiple reports have shown that NO can stimulate endogenous melatonin accumulation in sunflower seedling cotyledons following salt exposure, suggesting that NO partially regulates melatonin signaling [66,72]. These contradictory conclusions may be due to the different salt treatments and plant species used in the experiments.

#### 3.1.3. Ca^2+^

The concentration of Ca^2+^, a second messenger in many signal transduction pathways, usually increases in response to external stimuli, and activated Ca^2+^ channels have been reported to cause a rapid increase in cytosolic Ca^2+^ levels after exposure to NaCl [13,73].

Recent studies have shown a synergistic effect between NO and Ca^2+^ during salt exposure. Khan et al. [54] showed that the application of SNP and CaCl_2_ to salt-stressed mustard leaves prevented a rise in the H_2_O_2_ content and membrane damage, while the addition of SNP and Ca(NO_3_)_2_ promoted plant growth, chlorophyll content, and root vigor in salt-stressed wheat seedlings [74]. NO and Ca^2+^ effectively alleviate the adverse effects of salt stress as part of the antioxidant system and by maintaining ion homeostasis [74]. Similarly, NO interacts with Ca^2+^ in *A. corniculatum* to up-regulate the PM Na^+^/H^+^ antiporter system [75].

In addition to a synergistic effect, feedback regulation exists between NO and Ca^2+^. The role of Ca^2+^ in initiating NO production was examined. The results showed that the Ca^2+^/CaM complex interacts directly with GSNOR and inhibits its activity, thereby stimulating NO accumulation and ion homeostasis to confer salt resistance [13]. The idea that NO modulates the Ca^2+^ level in plant cells is supported by other observations. For instance, Khan et al. [54] found that the exposure of salt-stressed leaves to SNP raised the Ca^2+^ concentration, whereas leaf exposure to cPTIO lowered the Ca^2+^ concentration. Additionally, Lang et al. [76] showed that the stimulation of Ca^2+^-SOS signaling by NO promoted the efflux of Na^+^ in salt-stressed *Glycyrrhiza uralensis*. These results suggest the existence of feedback regulation between NO and Ca^2+^ in the salt signaling pathway.

#### 3.1.4. CO

CO, usually created by the incomplete combustion of organic materials, can produce asphyxia by reversibly combining with hemoglobin. It was recently found that CO enhances salt tolerance in wheat via an NO-mediated signaling pathway [77]. The application of a 50% CO-saturated aqueous solution enhanced the activity of PM H^+^-ATPase and antioxidant enzymes to boost salt tolerance. Salt-stressed seedlings treated with CO showed a rapid increase in endogenous NO at the root tips [77]. In contrast, treatment with cPTIO almost completely blocked CO-induced NO production [77]. These results suggest that CO can alleviate salinity-induced damage through a NO-dependent pathway.

#### 3.1.5. S

S is the fourth most important essential nutrient in plants, after N, phosphorus, and potassium, and its role alongside NO in preventing extensive salt-induced damage in plants has been well documented.

S and NO are emerging as messenger molecules involved in the regulation of salt stress processes in *Cyclocarya paliurus* and barley [78,79]. Moreover, treatment with NO and S reduced the contents of Na^+^ and Cl^−^ by regulating Na^+^ transporter and H^+^ pump function in leaves and roots [44]. Further, supplementation of NO and S to plants promoted the activity of the ROS-scavenging enzymes CAT, APX, and GR [44], and mustard leaves treated with NO plus S opened their stomata, thereby improving their photosynthetic performance [38,44]. These data indicate that S and NO have synergistic effects on plant salt tolerance.

There may also be an interaction between NO and S assimilation. NO was shown to increase S assimilation in plants, leading to an increase in the contents of cysteine and GSH to prevent oxidative damage due to salt stress [44,80]. Future studies should focus on the role of NO in regulating the S assimilation pathway and S metabolites under conditions of salt stress.

### 3.2. NO and Plant Hormones

Recent studies have revealed a close relationship between NO and plant hormones in plant salt stress responses and signal transduction.

#### 3.2.1. Auxin

Auxin, the first plant hormone discovered, plays an important role in plant development and growth as well as in the responses of plants to environmental salt stress.

One report showed that the foliar application of bioactive auxin, indole-3-acetic acid (IAA), improved the chlorophyll level and maintained membrane stability in salt-stressed maize plants [81]. These data suggest that auxin plays a positive role in plants under salt stress.

Meanwhile, a separate report showed that the application of NO or auxin alone could alleviate salt damage in *B. juncea* [82] but that the effect was more pronounced when both compounds were applied together. This suggests a synergistic effect between NO and auxin under salt stress [82].

NO was also reported to be an essential downstream signal in the IAA-induced tolerance of cucumber to salt stress [83]; however, Liu et al. [84] suggested that salt-induced NO functions upstream of auxin in *Arabidopsis*. In that study, NO lowered auxin levels by repressing the expression of the auxin efflux transporter gene *PIN* and reducing auxin signal transduction by stabilizing the Aux/IAA suppressor protein IAA17. As a result, root meristem growth was inhibited. It has also been reported that NO participates in tomato root development under saline conditions by changing the distribution of auxin [85].

In brief, current studies suggest that the growth hormone auxin and NO act synergistically under salt stress; however, the upstream and downstream relationships between them are unclear and require further investigation.

#### 3.2.2. Gibberellic Acid (GA)

GA is a classic growth-promoting phytohormone that modulates plant growth and development and regulates stress responses. GA promotes growth via the proteasome-mediated degradation of DELLA transcriptional repressor proteins. The *Arabidopsis della* double mutant was hypersensitive to salt treatment, indicating that GA played a negative role in salt responses [86]. Interaction between GA and NO in salt resistance has also been found in *Arabidopsis* [87,88].

Liu et al. [84] suggested that salt-induced NO stabilized RGL3, a DELLA protein, and IAA17, resulting in a lower level of bioactive GA_4_. Furthermore, IAA17 and RGL3 were shown to interact with and stabilize each other, resulting in enhanced salt stress resistance [87]. These data suggest that NO mediates salt stress responses through the integration of auxin and GA signaling.

Recently, Zuo et al. [88] found that NO negatively regulates GA signaling through the sulfhydryl nitrosylation (*S*-nitrosylation) of DELLA proteins to coordinate the balance between plant growth and salt stress responses. The detailed mechanism of *S*-nitrosylation will be addressed in the next section.

NO affects the salt sensitivity of plants by influencing the stability of DELLA proteins; thus, the crosstalk between NO and other components of the GA signaling pathway is worthy of further investigation.

#### 3.2.3. Cytokinin (CK)

CK plays a role in the response of plants to salt stress. For example, it regulates Na^+^ accumulation in the shoot by controlling the expression of *Arabidopsis high-affinity K^+^ transporter 1;1*, a gene responsible for removing Na^+^ from the root xylem. This regulation depends on such transcription factors as ARR1 and ARR12 in the CK signaling pathway [89].

A previous report showed that CK and NO regulated the salt tolerance of perennial ryegrass by reducing Na^+^ accumulation and improving growth and photochemical efficiency [90]. Other researchers demonstrated that the foliar application of SNP increased the expression of CK biosynthesis genes (e.g., *ZR*, *IPA*, and *IPT1*) in cotton, suggesting that NO promotes CK biosynthesis, which delays salt-induced leaf senescence [91]. Maslennikova et al. [92] also found a positive impact of NO on the CK content of wheat plants under salt stress.

These data suggest that NO positively affects the CK content of plants to promote salt resistance.

#### 3.2.4. Abscisic Acid (ABA)

ABA protects plants in response to various stressful stimuli, including salinity. Researchers found that both NaCl and ABA could induce NO production [93]. Ruan et al. [94] reported that exogenous SNP treatment dramatically activated the synthesis of endogenous ABA in salt-stressed wheat seedlings. Later reports revealed that NO is positively involved in the regulation of ABA accumulation under conditions of salt stress in rice and maize [95,96]. Further, NO reduces the expression of two ABA biosynthesis-related genes, *NCED2* and *NCED9*, to delay salt-induced leaf senescence in cotton [97]. These data suggest that ABA production is dependent on NO in salt-regulated root growth.

However, some researchers found that NO might work downstream of ABA. For example, *NCED* overexpression increased both the ABA content and salt tolerance of tobacco plants. This promoted the production of H_2_O_2_ by NADPH oxidase and NO by a NOS-like enzyme and increased both the transcript and activity levels of SOD, CAT, APX, and GR [98]. In addition, Santos et al. [85] observed that salt stress increased NO accumulation, H^+^-ATPase activity, and APX and CAT activity in wild-type tomatoes but not in the ABA-deficient *sitiens* mutant.

These studies indicate that the relationship between NO and ABA under salt stress is complicated.

#### 3.2.5. Ethylene

Ethylene, a gaseous hormone, is closely related to salt stress in plants. High-level accumulation of Na^+^ in plants under salt stress increased ethylene production [99], while a loss-of-function mutation in *EIN2*, a positive regulator of ethylene signaling, resulted in salt hypersensitivity [100]. Meanwhile, ethylene and NO accumulation were increased by 100 mM NaCl in the root apices and suspension culture cells of tomatoes [101]. Together, ethylene and NO stimulate PM H^+^-ATPase activity to modulate ion homeostasis and salt tolerance [102].

Furthermore, ethylene may be a downstream signaling molecule of NO in *Arabidopsis* [102]. In sunflowers, NO functions as a negative regulator of the ethylene biosynthesis-related protein ACC oxidase. NO depletion under salt stress was shown to enhance ACC oxidase activity, and the resulting drop in ethylene promoted lateral root formation [103]. According to Wang et al. [104], NO acts as an upstream signaling molecule in the ethylene-mediated induction of the *AOX* gene and pyruvate content, thereby inducing an alternative respiratory pathway and avoiding ROS damage in plant cells under salt stress. These data indicate that ethylene may be a part of the downstream signal molecular in NO action under salt stress.

Salinity increases ethylene emission, which in turn increases NO production, and ethylene acts at least in part through the NO pathway. NO and ethylene form a positive feedback loop in promoting seed germination by decreasing H_2_O_2_ level under salinity [105].

Regarding the relationship between ethylene and NO, it is difficult to define which one acts upstream of the other; it may depend on the physiological response.

#### 3.2.6. Brassinosteroids (BRs)

BRs are a new group of plant hormones with significant growth-promoting effects as well as roles in environmental stress responses.

One study showed that *Medicago sativa* seeds treated with a bioactive BR at a suitable concentration improved seed germination and seedling growth in saline soil, indicating that BRs play a positive regulatory role in the response of plants to salt stress [106].

Another report showed that BR- and NO-mediated increases in plant adaptation to salinity stress are tied to their impact on N, proline, and ABA metabolism [52].

Zhu et al. [107] found that *Nicotiana benthamiana* seedlings pretreated with BRs showed greater tolerance to salt stress, accompanied by an increase in cyanide-resistant respiration. AOX plays an important role in ROS scavenging in plant mitochondria. Pretreatment with BRs alleviated salt-induced oxidative damage and increased the *AOX1a* transcript level, which depends on NO biosynthesis [107]. These data indicate that NO is involved in BR-induced AOX activity, which plays an essential role in salt tolerance in *N. benthamiana* seedlings.

#### 3.2.7. Salicylic Acid (SA)

SA is a phenolic compound found widely in higher plants that participates in the regulation of plant physiology, systemic plant defenses, and plant responses to biotic/abiotic stresses. Studies indicate that SA and NO act synergistically to decrease the deleterious effects of salt stress.

Khan et al. [108] demonstrated that SA increased glycine betaine accumulation to protect the photosynthetic system in mung beans under salt stimulation. It is also been suggested that NO is involved in H_2_O_2_- and SA-induced reductions in oxidative damage in rice and cotton through the upregulation of antioxidant defenses and methylglyoxal detoxification systems [109,110].

When SA or NO was applied to salt-stressed *V. angularis*, organelle damage was prevented by the accumulation of proline, glycine betaine, and sugar. Further, the exogenous application of SA and NO improved the growth performance and photosynthetic efficiency of *V. angularis* by reducing oxidative stress [67]. Consistent with these findings, the application of SA and NO improved growth and biomass accumulation in salt-stressed *V. angularis* and *Gossypium hirsutum* [67,111].

In summary, NO and SA work synergistically to improve osmoregulation and antioxidant system function to maintain a normal metabolism.

#### 3.2.8. Jasmonic Acid (JA)

JA plays a critical role in limiting damage due to abiotic stresses, and JA biosynthesis and signaling pathways are affected by NO [112].

Ahmad et al. [51] showed that JA and NO, applied either individually or in combination, up-regulated antioxidant metabolism, osmolyte synthesis, and metabolite accumulation. Yastreb et al. [113] reported that a defect in JA signaling (*jin1/myc2*) compromised the NO-dependent induction of some plant defensive responses to salt stress in *Arabidopsis*.

In brief, NO and JA enhance the salt tolerance of seedlings by protecting the osmotic balance and redox homeostasis. However, the exact mechanism by which JA and NO mediate Na^+^ signaling is unclear.

The above studies suggest that NO interacts with other signaling molecules to reduce or mitigate the harmful effects of salt stress on plant metabolism and development (Table 1). The combined application of NO and various signaling molecules effectively modulates salt stress tolerance by stimulating osmolyte accumulation and the activities of enzymatic and non-enzymatic antioxidants to reduce ROS production and lipid peroxidation. However, the regulation of plant responses to salt stress often involves complex signaling cascades integrating multiple environmental and developmental inputs. Therefore, additional studies are needed to examine the crosstalk between NO and various signaling molecules in salt stress responses to fine-tune the growth, development, and metabolism of plants under high-salt conditions.

## 4. Molecular Mechanisms of NO Signaling

**Figure 2:**
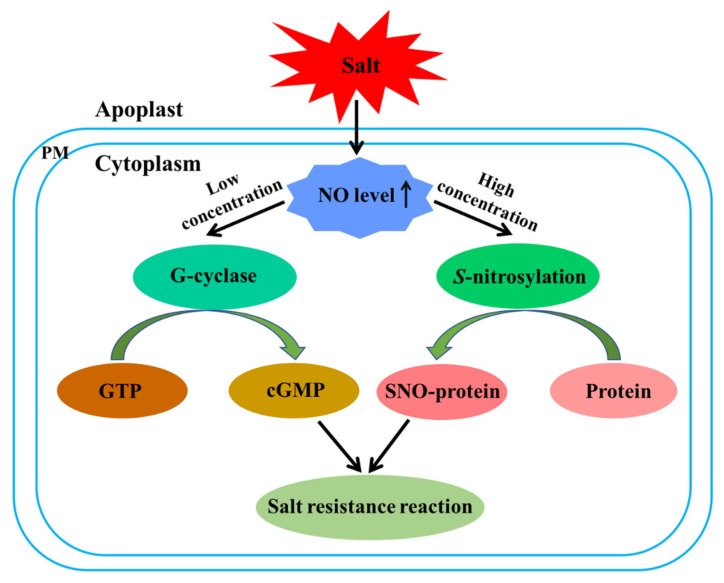
The molecular mechanisms of NO-mediated salt tolerance in plants.

NO, a crucial signaling molecule, plays a central role in plant resistance to salt stress. Similar to the NO signaling pathway in animals, cyclic guanosine monophosphate (cGMP)-dependent and -independent pathways exist. The cGMP-dependent pathway takes place at low NO concentrations and is initiated through the activation of soluble guanylate cyclase, which catalyzes the conversion of GTP to cGMP. Studies of the cGMP-independent pathway have led to the identification of numerous *S*-nitrosylated proteins, including key regulatory/signaling proteins that control both growth and stress responses in plants (e.g., RGA in crosstalk between GA and salt signaling and OST1 and ABI5 in ABA signaling) [88,114,115] (Figure 2).

### 4.1. cGMP

Evidence shows that the NO-cGMP signaling pathway is important in the activation of defensive responses to salt stress [116]. Exogenous application of the antioxidant caffeic acid stimulated the NO-cGMP cascade in soybean root nodules, resulting in the scavenging of ROS to reduce salinity-induced oxidative stress. This phenomenon suggested the involvement of NO-cGMP signaling in plant responses to salinity. However, the downstream targets of the NO-cGMP signaling pathway are unknown.

### 4.2. Protein S-Nitrosylation

NO performs its physiological role primarily through protein *S*-nitrosylation, a redox-based posttranslational modification. Protein *S*-nitrosylation is an evolutionarily conserved mechanism that regulates multiple aspects of cellular signaling. *S*-Nitrosothiols are formed by combining NO with cysteine residues; this alters the characteristics of the modified proteins, including their enzymatic activity, subcellular localization, stability, and protein–protein interactions.

Tanou et al. [117] found 49 differentially *S*-nitrosylated proteins in NaCl-treated citrus leaves. Meanwhile, NaCl treatment resulted in enhanced *S*-nitrosylation in tobacco, sunflower seedlings, and tomatoes [118,119,120]. In contrast, the signal for *S*-nitrosylation was reduced in peas and citrus in response to salt treatment [121,122]. These inconsistent results regarding *S*-nitrosylated protein levels under salt stress may be due to differences in the plant species and tissues analyzed.

A previous study showed that the *S*-nitrosylation of Cys-374 in *Arabidopsis* RGA, a DELLA protein, prevented its degradation by the proteasome under saline conditions. The consequent accumulation of RGA retards growth but enhances salt tolerance, so it has been proposed that NO negatively regulates GA signaling via the *S*-nitrosylation of RGA to coordinate the balance between growth and stress responses [88].

Our laboratory has made some progress in uncovering the role of NO in plant salt tolerance. In 2004, we discovered that NO served as a signal in inducing salt resistance by increasing the K^+^/Na^+^ ratio in reed calli [6]. Follow-up work then proposed that Ca^2+^ stimulates NO production in a high-salt environment [13]. However, there are still questions that need answering. Research by our group and others on the regulation of salt tolerance by NO is ongoing. At present, several *S*-nitrosylated proteins are being screened; the work may provide new insight into the molecular mechanisms underlying plant responses to salt stress.

## 5. Conclusions and Perspectives

Over the past two decades, research has shown that NO is a “star molecule” in mediating plant salt tolerance. Most work on NO signaling in salt-stressed plants has focused on NO production, its ability to limit salt-induced damage to plants during growth and development, its relationship with other signaling molecules, and its regulatory mechanism. However, the understanding of the molecular role of NO in plants following salt exposure is incomplete. Future studies should focus on the following areas: (1) A primary NO signaling pathway should be established for salt resistance by integrating several existing fragmentary pathways together. (2) The precise molecular mechanism underlying the effect of NO on salt tolerance in plants should be explored; this will necessitate identifying additional *S*-nitrosylated proteins. (3) Apart from protein *S*-nitrosylation, other mechanisms whereby NO may regulate plant growth and development under salt stress should be studied. For example, denitrosylation and transnitrosylation were recently discovered, and they may affect plant responses to salinity. Further, the continuous application of newly available sophisticated methodologies, including genome-wide expression analysis, proteomics, and metabolomics, will help identify novel NO-responsive genes and protein and metabolite networks and contribute to the generation of new hypotheses. Many questions could be answered with further advancements in biological technology.

## Figures and Tables

**Table 1:** Effects of NO and different signaling molecules under salt stress.

Signal Molecule	Origen	Method of Application	Position/Stage	Major Effects/Response	References
Polyamines (PA)	Sunflower	DETA	Cotyledons	Upregulates PA biosynthetic enzymes and reduces the activity of PA oxidase	[69]
	Cucumber	SNP	Leaves and roots	Regulates the free PA content	[70]
Melatonin	Sunflower	SNP + Melatonin	Cotyledons	Modulates GR activity	[66]
	Rapeseed		Roots	Decreases the Na⁺/K⁺ ratio Reduces reactive oxygen overproduction	[71]
Calcium ions (Ca^2+^)	Mustard/ Wheat	SNP + CaCl_2_/ SNP + Ca(NO_3_)_2_	Leaves/ Seedlings	Alleviates a decline in chlorophyll content Decreases electrolyte leakage Enhances the proline content and glycine betaine accumulation Decreases the H_2_O_2_ content Enhances the activities of antioxidative enzymes	[54,74]
Carbon monoxide (CO)	Wheat	SNP/DETA + 50% CO aqueous solution	Roots	Up-regulates H^+^-pumps Maintains ion homeostasis Enhances the antioxidant system	[77]
Sulfur (S)	Mustard	SNP/DETA + Sulphate	Leaves	Increases proline	[38]
		SNP + Diallyl monosulfide (DAS)		Regulates chloroplast development	[44]
		SNP/DETA + Sulphate/ SNP + DAS		Promotes photosynthesis Increases antioxidant system activity Affects S assimilation	[38,44]
Auxin	*Brassica juncea*	NO + IAA	Leaves	Promotes photosynthetic efficiency Enhances proline accumulation Enhances antioxidant system activity	[82]
	Arabidopsis	SNP	Roots	Represses the expression of gene *PIN* Stabilizes the protein IAA17	[84]
Gibberellic acid (GA)	Arabidopsis	SNP	Seedlings	Stabilizes RGL3	[87]
		SNP/GSNO		Inhibits proteasome degradation through *S*-nitrosylation	[88]
Cytokinin (CK)	*Lolium* *perenne*	SNP + 6-Benzyladenine	Leaves	Improves growth Improves photochemical efficiency Reduces Na^+^ accumulation	[90]
	Cotton	SNP		Increases cytokinin biosynthesis gene expression	[91]
Abscisic acid (ABA)	Tomato	SNP	Roots	Regulates H^+^-ATPase activity Regulates antioxidative enzyme activities	[85]
	Wheat		Leaves	Activates the synthesis of endogenous ABA	[94,95,96]
	Rice		Seeds		
	Maize		Seedlings		
Ethylene	Arabidopsis	SNP/1-aminocyclopropane-1-carboxylic acid	Callus	Stimulates PM H^+^-ATPase activity	[102]
				Increases *AOX1* expression Enhances pyruvate content	[104]
		SNP	Seeds	Induces the expression of *ACS2* involved in ethylene synthesis	[105]
Brassinosteroids (BR)	*Brassica juncea*	SNP + 24-Epibrassinolide	Leaves	Limits the Na^+^ accumulation Increases photosynthetic traits Increases proline Improves nitrogen metabolism	[52]
	*Nicotiana* *benthamiana*	SNP	Seedlings	Improves alternative pathway respiration	[107]
Salicylic acid (SA)	Cotton	SNP + SA	Seedlings	Alleviates the inhibition of H^+^-ATPase	[110]
	*Vigna angularis* /Cotton			Improves uptake of mineral nutrients Increases photosynthesis Activates the metabolic of osmotic-regulated substances Improves antioxidant system	[67,110,111]
Jasmonic acid (JA)	Tomato	SNAP + JA	Seedlings	Up-regulates antioxidant metabolism Boosts metabolite accumulation	[51,113]
	Arabidopsis	SNP			

## Data Availability

Not applicable.

## References

[1] Moreau M., Lee G.I., Wang Y., Crane B.R., Klessig D.F. (2008). AtNOS/AtNOA1 is a functional *Arabidopsis thaliana* cGTPase and not a nitric oxide synthase. J. Biol. Chem..

[2] Corpas F.J., Palma J.M., Del Río L.A., Barroso J.B. (2009). Evidence supporting the existence of L-arginine-dependent nitric oxide synthase activity in plants. N. Phytol..

[3] Astier J., Gross I., Durner J. (2018). Nitric oxide production in plants: An update. J. Exp. Bot..

[4] Ventimiglia L., Mutus B. (2020). The physiological implications of *S*-nitrosoglutathione reductase (GSNOR) activity mediating no signalling in plant root structures. Antioxidants.

[5] Hasanuzzaman M., Oku H., Nahar K., Bhuyan M.H.M.B., Al Mahmud J., Baluska F., Fujita M. (2018). Nitric oxide-induced salt stress tolerance in plants: ROS metabolism, signaling, and molecular interactions. Plant Biotechnol. Rep..

[6] Zhao L., Zhang F., Guo J., Yang Y., Li B., Zhang L. (2004). Nitric oxide functions as a signal in salt resistance in the calluses from two ecotypes of reed. Plant Physiol..

[7] Cai W., Liu W., Wang W.S., Fu Z.W., Han T.T., Lu Y.T. (2015). Overexpression of rat neurons nitric oxide synthase in rice enhances drought and salt tolerance. PLoS ONE.

[8] Kaya C., Ashraf M. (2021). Nitric oxide is required for aminolevulinic acid-induced salt tolerance by lowering oxidative stress in maize (*Zea mays*). J. Plant Growth Regul..

[9] Zhao M.G., Tian Q.Y., Zhang W.H. (2007). Nitric oxide synthase-dependent nitric oxide production is associated with salt tolerance in Arabidopsis. Plant Physiol..

[10] Reda M., Golicka A., Kabala K., Janicka M. (2018). Involvement of NR and PM-NR in NO biosynthesis in cucumber plants subjected to salt stress. Plant Sci..

[11] Liu Y., Wu R., Wan Q., Me G., Bi Y. (2007). Glucose-6-phosphate dehydrogenase plays a pivotal role in nitric oxide-involved defense against oxidative stress under salt stress in red kidney bean. Plant Cell Physiol..

[12] Xie Y., Mao Y., Lai D., Zhang W., Zheng T., Shen W. (2013). Roles of NIA/NR/NOA1-dependent nitric oxide production and HY1 expression in the modulation of *Arabidopsis* salt tolerance. J. Exp. Bot..

[13] Zhou S., Jia L., Chu H., Wu D., Peng X., Liu X., Zhang J., Zhao J., Chen K., Zhao L. (2016). *Arabidopsis* CaM1 and CaM4 promote nitric oxide production and salt resistance by inhibiting *S*-nitrosoglutathione reductase via direct binding. PLoS Genet..

[14] Sun X., Zhuang Y., Lin H., Zhou H. (2019). Patellin1 negatively regulates plant salt tolerance by attenuating nitric oxide accumulation in Arabidopsis. Plant Signal. Behav..

[15] Yin X., Hu Y., Zhao Y., Meng L., Zhang X., Liu H., Wang L., Cui G. (2021). Effects of exogenous nitric oxide on wild barley (*Hordeum brevisubulatum*) under salt stress. Biotechnol. Biotechnol. Equip..

[16] Gao Z., Zhang J., Zhang J., Zhang W., Zheng L., Borjigin T., Wang Y. (2022). Nitric oxide alleviates salt-induced stress damage by regulating the ascorbate-glutathione cycle and Na^+^/K^+^ homeostasis in *Nitraria tangutorum* Bobr. Plant Physiol. Biochem..

[17] Yang Y., Guo Y. (2018). Elucidating the molecular mechanisms mediating plant salt-stress responses. N. Phytol..

[18] Zhu J. (2003). Regulation of ion homeostasis under salt stress. Curr. Plant Biol..

[19] Amin I., Rasool S., Mir M.A., Wani W., Masoodi K.Z., Ahmad P. (2021). Ion homeostasis for salinity tolerance in plants: A molecular approach. Physiol. Plant..

[20] Hasanuzzaman M., Inafuku M., Nahar K., Fujita M., Oku H. (2021). Nitric oxide regulates plant growth, physiology, antioxidant defense, and ion homeostasis to confer salt tolerance in the mangrove species, *Kandelia obovata*. Antioxidants.

[21] Ren Y., Wang W., He J., Zhang L., Wei Y., Yang M. (2020). Nitric oxide alleviates salt stress in seed germination and early seedling growth of pakchoi (*Brassica chinensis* L.) by enhancing physiological and biochemical parameters. Ecotoxicol. Environ. Saf..

[22] Dong Y., Zhang Q., Dai X., He M. (2020). Effects of potassium chloride and nitric oxide on growth and physiological characteristics of winter wheat under salt stress. Biol. Plant..

[23] Habib N., Ashraf M. (2014). Effect of exogenously applied nitric oxide on water relations and ionic composition of rice (*Oryza sativa* L.) plants under salt stress. Pak. J. Bot..

[24] Vaishnav A., Jain S., Kasotia A., Kumari S., Gaur R.K., Choudhary D.K. (2013). Effect of nitric oxide signaling in bacterial-treated soybean plant under salt stress. Arch. Microbiol..

[25] Ruan H., Shen W., Xu L. (2004). Nitric oxide modulates the activities of plasma membrane H^+^-ATPase and PPase in wheat seedling roots and promotes the salt tolerance against salt stress. Acta Bot. Sin..

[26] Chen J., Xiao Q., Wu F., Dong X., He J., Pei Z., Zheng H. (2010). Nitric oxide enhances salt secretion and Na^+^ sequestration in a mangrove plant, *Avicennia marina*, through increasing the expression of H^+^-ATPase and Na^+^/H^+^ antiporter under high salinity. Tree Physiol..

[27] Chen J., Xiong D.Y., Wang W.H., Hu W.J., Simon M., Xiao Q., Chen J., Xiong D.Y., Wang W.H., Hu W.H. (2013). Nitric oxide mediates root K^+^/Na^+^ balance in a mangrove plant, *Kandelia obovata*, by enhancing the expression of AKT1-Type K^+^ channel and Na^+^/H^+^ antiporter under high salinity. PLoS ONE.

[28] Zhang Y., Wang L., Liu Y., Zhang Q., Wei Q., Zhang W. (2006). Nitric oxide enhances salt tolerance in maize seedlings through increasing activities of proton-pump and Na^+^/H^+^ antiport in the tonoplast. Planta.

[29] Ding F. (2013). Effects of salinity and nitric oxide donor sodium nitroprusside (SNP) on development and salt secretion of salt glands of *Limonium bicolor*. Acta Physiol. Plant..

[30] Pereira T.M., dos Santos H.O., Neto A.R.D., Pelissari F., Pereira W.V., de Melo L.A. (2020). Does nitric oxide protect *Eucalyptus urophylla* seeds under salt stress conditions?. J. Seed Sci..

[31] Duan P., Ding F., Wang F., Wang B.S. (2007). Priming of seeds with nitric oxide donor sodium nitroprusside (SNP) alleviates the inhibition on wheat seed germination by salt stress. J. Plant Physiol. Mol. Biol..

[32] Habib N., Ashraf M., Ahmad M.S.A. (2010). Enhancement in seed germinability of rice (*Oryza sativa* L.) by pre-sowing seed treatment with nitric oxide (NO) under salt stress. Pak. J. Bot..

[33] Huang J., Zhu C., Hussain S., Huang J., Liang Q., Zhu L., Cao X., Kong Y., Li Y., Wang L. (2020). Effects of nitric oxide on nitrogen metabolism and the salt resistance of rice (*Oryza sativa* L.) seedlings with different salt tolerances. Plant Physiol. Biochem..

[34] Shams M., Ekinci M., Ors S., Turan M., Agar G., Kull R., Yildirim E. (2019). Nitric oxide mitigates salt stress effects of pepper seedlings by altering nutrient uptake, enzyme activity and osmolyte accumulation. Physiol. Mol. Biol. Plants.

[35] Chaves M.M., Flexas J., Pinheiro C. (2009). Photosynthesis under drought and salt stress: Regulation mechanisms from whole plant to cell. Ann. Bot..

[36] Papadakis I., Sotiras M.I., Landi M., Ladikou E., Oikonomou A., Psychoyou M., Fasseas C. (2019). Salinity alters plant’s allometry and sugar metabolism, and impairs the photosynthetic process and photosystem II efficiency in *Eriobotrya japonica* plants. Agrochimica.

[37] Wu X.X., Ding H.D., Chen J.L., Zhang H.J., Zhu W.M. (2010). Attenuation of salt-induced changes in photosynthesis by exogenous nitric oxide in tomato (*Lycopersicon esculentum* Mill. L.) seedlings. Afr. J. Biotechnol..

[38] Jahan B., AlAjmi M.F., Rehman M.T., Khan N.A. (2020). Treatment of nitric oxide supplemented with nitrogen and sulfur regulates photosynthetic performance and stomatal behavior in mustard under salt stress. Physiol. Plant..

[39] Sami F., Siddiqui H., Alam P., Hayat S. (2021). Nitric oxide mitigates the salt-induced oxidative damage in mustard by upregulating the activity of various enzymes. J. Plant Growth Regul..

[40] Wu X., Zhu X., Chen J., Yang S., Ding H., Zha D. (2013). Nitric oxide alleviates adverse salt-induced effects by improving the photosynthetic performance and increasing the anti-oxidant capacity of eggplant (*Solanum melongena* L.). J. Hortic. Sci. Biotechnol..

[41] Dadasoglu E., Ekinci M., Kul R., Shams M., Turan M., Yildirim E. (2021). Nitric oxide enhances salt tolerance through regulating antioxidant enzyme activity and nutrient uptake in pea. Legume Res..

[42] Liu A., Fan J.B., Gitau M.M., Chen L., Fu J. (2016). Nitric oxide involvement in bermudagrass response to salt stress. J. Am. Soc. Hortic. Sci..

[43] Yuezbasioglu E.A., Oz G.C. (2010). Relationship between salt stress and nitric oxide in *Glycine max* L. (soybean). Fresenius Environ. Bull..

[44] Fatma M., Masood A., Per T.S., Khan N.A. (2016). Nitric oxide alleviates salt stress inhibited photosynthetic performance by interacting with sulfur assimilation in mustard. Front. Plant Sci..

[45] Mehar F., Khan N. (2014). Nitric oxide protects photosynthetic capacity inhibition by salinity in Indian mustard. J. Funct. Environ. Bot..

[46] Shen Z.J., Chen J., Ghoto K., Hu W.J., Gao G.F., Luo M.R., Li Z., Simon M., Zhu X.Y., Zheng H.L. (2018). Proteomic analysis on mangrove plant *Avicennia marina* leaves reveals nitric oxide enhances the salt tolerance by up-regulating photosynthetic and energy metabolic protein expression. Tree Physiol..

[47] Alnusairi G.S.H., Mazrou Y.S.A., Qari S.H., Elkelish A.A., Soliman M.H., Eweis M., Abdelaal K., El-Samad G.A., Ibrahim M.F.M., ElNahhas N. (2021). Exogenous nitric oxide reinforces photosynthetic efficiency, osmolyte, mineral uptake, antioxidant, expression of stress-responsive genes and ameliorates the effects of salinity stress in wheat. Plants.

[48] Jian W., Zhang D.W., Zhu F., Wang S.X., Pu X.J., Deng X.G., Luo S.S., Lin H.H. (2016). Alternative oxidase pathway is involved in the exogenous SNP-elevated tolerance of *Medicago truncatula* to salt stress. J. Plant Physiol..

[49] Zhao S., Zhang Q., Liu M., Zhou H., Ma C., Wang P. (2021). Regulation of plant responses to salt stress. Int. J. Mol. Sci..

[50] Guo Y., Tian Z., Yan D., Zhang J., Qin P. (2009). Effects of nitric oxide on salt stress tolerance in *Kosteletzkya virginica*. Life Sci. J..

[51] Ahmad P., Ahanger M.A., Alyemeni M.N., Wijaya L., Alam P., Ashraf M. (2018). Mitigation of sodium chloride toxicity in *Solanum lycopersicum* L. by supplementation of jasmonic acid and nitric oxide. J. Plant Interact..

[52] Gupta P., Srivastava S., Seth C.S. (2017). 24-Epibrassinolide and sodium nitroprusside alleviate the salinity stress in *Brassica juncea* L. cv. Varuna through cross talk among proline, nitrogen metabolism and abscisic acid. Plant Soil.

[53] Wu X., Zhu W., Zhang H., Ding H., Zhang H. (2011). Exogenous nitric oxide protects against salt-induced oxidative stress in the leaves from two genotypes of tomato (*Lycopersicom esculentum* Mill.). Acta Physiol. Plant..

[54] Khan M.N., Siddiqui M.H., Mohammad F., Naeem M. (2012). Interactive role of nitric oxide and calcium chloride in enhancing tolerance to salt stress. Nitric Oxide.

[55] Ahmad P., Latef A.A.A., Hashem A., Abd Allah E.F., Gucel S., Tran L.S.P. (2016). Nitric oxide mitigates salt stress by regulating levels of osmolytes and antioxidant enzymes in chickpea. Front. Plant Sci..

[56] Sachdev S., Ansari S.A., Ansari M.I., Fujita M., Hasanuzzaman M. (2021). Abiotic stress and reactive oxygen species: Generation, signaling, and defense mechanisms. Antioxidants.

[57] Hasanuzzaman M., Bhuyan M.H.M.B., Parvin K., Bhuiyan T.F., Anee T.I., Nahar K., Hossen M.S., Zulfiqar F., Alam M.M., Fujita M. (2020). Regulation of ROS metabolism in plants under environmental stress: A review of recent experimental evidence. Int. J. Mol. Sci..

[58] Du S.T., Liu Y., Zhang P., Liu H.J., Zhang X.Q., Zhang R.R. (2015). Atmospheric application of trace amounts of nitric oxide enhances tolerance to salt stress and improves nutritional quality in spinach (*Spinacia oleracea* L.). Food Chem..

[59] Li Q.Y., Niu H.B., Yin J., Wang M.B., Shao H.B., Deng D.Z., Chen X.X., Ren J.P., Li Y.C. (2008). Protective role of exogenous nitric oxide against oxidative-stress induced by salt stress in barley (*Hordeum vulgare*). Coll. Surf. B.

[60] Liu J., Fu C., Li G., Khan M., Wu H. (2021). ROS homeostasis and plant salt tolerance: Plant nanobiotechnology updates. Sustainability.

[61] Egbichi I., Keyster M., Jacobs A., Klein A., Ludidi N. (2013). Modulation of antioxidant enzyme activities and metabolites ratios by nitric oxide in short-term salt stressed soybean root nodules. S. Afr. J. Bot..

[62] Chen J., Xiao Q., Wang C., Wang W.H., Wu F.H., Chen J., He B.Y., Zhu Z., Ru Q.M., Zhang L.L. (2014). Nitric oxide alleviates oxidative stress caused by salt in leaves of a mangrove species, *Aegiceras corniculatum*. Aquat. Bot..

[63] Liu S., Dong Y., Xu L., Kong J., Bai X. (2013). Roles of exogenous nitric oxide in regulating ionic equilibrium and moderating oxidative stress in cotton seedlings during salt stress. J. Soil Sci. Plant Nutr..

[64] Karami A., Sepehri A. (2018). Nano titanium dioxide and nitric oxide alleviate salt induced changes in seedling growth, physiological and photosynthesis attributes of barley. Zemdirbyste.

[65] Liu H., Li C., Yan M., Zhao Z., Huang P., Wei L., Wu X., Wang C., Liao W. (2022). Strigolactone is involved in nitric oxide-enhanced the salt resistance in tomato seedlings. J. Plant Res..

[66] Kaur H., Bhatla S.C. (2016). Melatonin and nitric oxide modulate glutathione content and glutathione reductase activity in sunflower seedling cotyledons accompanying salt stress. Nitric Oxide.

[67] Ahanger M.A., Aziz U., Alsahli A.A., Alyemeni M.N., Ahmad P. (2020). Influence of exogenous salicylic acid and nitric oxide on growth, photosynthesis, and ascorbate-glutathione cycle in salt stressed *Vigna angularis*. Biomolecules.

[68] Locato V., Paradiso A., Sabetta W., De Gara L., de Pinto M.C. (2016). Nitric oxide and reactive oxygen species in PCD signaling. Adv. Bot. Res..

[69] Tailor A., Tandon R., Bhatla S.C. (2019). Nitric oxide modulates polyamine homeostasis in sunflower seedling cotyledons under salt stress. Plant Signal. Behav..

[70] Fan H.F., Du C.X., Guo S.R. (2013). Nitric oxide enhances salt tolerance in cucumber seedlings by regulating free polyamine content. Environ. Exp. Bot..

[71] Zhao G., Zhao Y., Yu X., Kiprotich F., Han H., Guan R., Wang R., Shen W. (2018). Nitric oxide is required for melatonin-enhanced tolerance against salinity stress in rapeseed (*Brassica napus* L.) seedlings. Int. J. Mol. Sci..

[72] Arora D., Bhatla S.C. (2017). Melatonin and nitric oxide regulate sunflower seedling growth under salt stress accompanying differential expression of Cu/Zn SOD and Mn SOD. Free Radic. Biol. Med..

[73] Choi W.G., Toyota M., Kim S.H., Hilleary R., Gilroy S. (2014). Salt stress-induced Ca^2+^ waves are associated with rapid, long-distance root-to-shoot signaling in plants. Proc. Natl. Acad. Sci. USA.

[74] Tian X., He M., Wang Z., Zhang J., Song Y., He Z., Dong Y. (2015). Application of nitric oxide and calcium nitrate enhances tolerance of wheat seedlings to salt stress. Plant Growth Regul..

[75] Lang T., Sun H., Li N., Lu Y., Shen Z., Jing X., Xiang M., Shen X., Chen S. (2014). Multiple signaling networks of extracellular ATP, hydrogen peroxide, calcium, and nitric oxide in the mediation of root ion fluxes in secretor and non-secretor mangroves under salt stress. Aquat. Bot..

[76] Lang T., Deng S., Zhao N., Deng C., Zhang Y., Zhang Y., Zhang H., Sa G., Yao J., Wu C. (2017). Salt-sensitive signaling networks in the mediation of K^+^/Na^+^ homeostasis gene expression in *Glycyrrhiza uralensis* roots. Front. Plant Sci..

[77] Xie Y., Ling T., Han Y., Liu K., Zheng Q., Huang L., Yuan X., He Z., Hu B., Fang L. (2008). Carbon monoxide enhances salt tolerance by nitric oxide-mediated maintenance of ion homeostasis and up-regulation of antioxidant defence in wheat seedling roots. Plant Cell Environ..

[78] Chen P., Yang W., Jin S., Liu Y. (2021). Hydrogen sulfide alleviates salinity stress in *Cyclocarya paliurus* by maintaining chlorophyll fluorescence and regulating nitric oxide level and antioxidant capacity. Plant Physiol. Biochem..

[79] Chen J., Wang W.H., Wu F.H., He E.M., Liu X., Shangguan Z.P., Zheng H.L. (2015). Hydrogen sulfide enhances salt tolerance through nitric oxide-mediated maintenance of ion homeostasis in barley seedling roots. Sci. Rep..

[80] Fatma M., Masood A., Per T.S., Rasheed F., Khan N.A. (2016). Interplay between nitric oxide and sulfur assimilation in salt tolerance in plants. Crop J..

[81] Kaya C., Tuna A.L., Dikilitas M., Cullu M.A. (2010). Responses of some enzymes and key growth parameters of salt-stressed maize plants to foliar and seed applications of kinetin and indole acetic acid. J. Plant Nutr..

[82] Shiraz M., Sami F., Siddiqui H., Yusuf M., Hayat S. (2020). Interaction of auxin and nitric oxide improved photosynthetic efficiency and antioxidant system of *Brassica juncea* plants under salt stress. J. Plant Growth Regul..

[83] Gong B., Miao L., Kong W., Bai J.G., Wang X., Wei M., Shi Q. (2014). Nitric oxide, as a downstream signal, plays vital role in auxin induced cucumber tolerance to sodic alkaline stress. Plant Physiol. Biochem..

[84] Liu W., Li R.J., Han T.T., Cai W., Fu Z.W., Lu Y.T. (2015). Salt stress reduces root meristem size by nitric oxide-mediated modulation of auxin accumulation and signaling in Arabidopsis. Plant Physiol..

[85] Santos M.P., Zandonadi D.B., de Sa A.F.L., Costa E.P., de Oliveira C.J.L., Perez L.E.P., Facanha A.R., Bressan-Smith R. (2020). Abscisic acid-nitric oxide and auxin interaction modulates salt stress response in tomato roots. Theor. Exp. Plant Physiol..

[86] Achard P., Cheng H., De Grauwe L., Decat J., Schoutteten H., Moritz T., Van Der Straeten D., Peng J.R., Harberd N.P. (2006). Integration of plant responses to environmentally activated phytohormonal signals. Science.

[87] Shi H., Liu W., Wei Y., Ye T. (2017). Integration of auxin/indole-3-acetic acid 17 and RGA-LIKE3 confers salt stress resistance through stabilization by nitric oxide in Arabidopsis. J. Exp. Bot..

[88] Chen L., Sun S., Song C.P., Zhou J.M., Li J., Zuo J. (2022). Nitric oxide negatively regulates gibberellin signaling to coordinate growth and salt tolerance in Arabidopsis. J. Genet. Genom..

[89] Mason M.G., Jha D., Salt D.E., Tester M., Hill K., Kieber J.J., Schaller G.E. (2010). Type-B response regulators ARR1 and ARR12 regulate expression of *AtHKT1;1* and accumulation of sodium in Arabidopsis shoots. Plant J..

[90] Ji Z., Camberato J.J., Zhang C., Jiang Y. (2019). Effects of 6-benzyladenine, gamma-aminobutyric acid, and nitric oxide on plant growth, photochemical efficiency, and ion accumulation of perennial ryegrass cultivars to salinity stress. Hortscience.

[91] Kong X., Wang T., Li W., Tang W., Zhang D., Dong H. (2016). Exogenous nitric oxide delays salt-induced leaf senescence in cotton (*Gossypium hirsutum* L.). Acta Physiol. Plant..

[92] Maslennikova D.R., Allagulova C.R., Fedorova K.A., Plotnikov A.A., Avalbaev A.M., Shakirova F.M. (2017). Cytokinins contribute to realization of nitric oxide growth-stimulating and protective effects on wheat plants. Russ. J. Plant Physiol..

[93] Hsu Y., Lee T.M. (2018). Abscisic acid-dependent nitric oxide pathway and abscisic acid-independent nitric oxide routes differently modulate NaCl stress induction of the gene expression of methionine sulfoxide reductase A and B in rice roots. J. Plant Physiol..

[94] Ruan H., Shen W., Xu L. (2004). Nitric oxide involved in the abscisic acid induced proline accumulation in wheat seedling leaves under salt stress. Acta Bot. Sin..

[95] Yi Y., Peng Y., Song T., Lu S., Teng Z., Zheng Q., Zhao F., Meng S., Liu B., Peng Y. (2022). NLP2-NR module associated NO is involved in regulating seed germination in rice under salt stress. Plants.

[96] Chen K., Li J., Tang J., Zhao F.G., Liu X. (2006). Involvement of nitric oxide in regulation of salt stress-induced ABA accumulation in maize seedling. J. Plant Physiol. Mol. Biol..

[97] Kong X., Luo Z., Dong H., Eneji A.E., Li W., Lu H. (2013). Gene expression profiles deciphering leaf senescence variation between early- and late-senescence cotton lines. PLoS ONE.

[98] Zhang Y., Tan J., Guo Z., Lu S., He S., Shu W., Zhou B. (2009). Increased abscisic acid levels in transgenic tobacco over-expressing 9 cis-epoxycarotenoid dioxygenase influence H_2_O_2_ and NO production and antioxidant defences. Plant Cell Environ..

[99] Zhao X., Schaller G.E. (2004). Effect of salt and osmotic stress upon expression of the ethylene receptor ETR1 in *Arabidopsis thaliana*. FEBS Lett..

[100] Lei G., Shen M., Li Z.G., Zhang B., Duan K.X., Wang N., Cao Y.R., Zhang W.K., Ma B., Ling H.Q. (2011). EIN2 regulates salt stress response and interacts with a MA3 domain-containing protein ECIP1 in *Arabidopsis*. Plant Cell Environ..

[101] Poor P., Borbely P., Kovacs J., Pap A., Szepesi A., Takacs Z., Tari I. (2014). Opposite extremes in ethylene/nitric oxide ratio induce cell death in suspension culture and root apices of tomato exposed to salt stress. Acta Biol. Hung..

[102] Wang H., Liang X., Wan Q., Wang X., Bi Y. (2009). Ethylene and nitric oxide are involved in maintaining ion homeostasis in *Arabidopsis* callus under salt stress. Planta.

[103] Singh N., Bhatla S.C. (2018). Nitric oxide regulates lateral root formation through modulation of ACC oxidase activity in sunflower seedlings under salt stress. Plant Signal. Behav..

[104] Wang H., Huang J., Bi Y. (2010). Induction of alternative respiratory pathway involves nitric oxide, hydrogen peroxide and ethylene under salt stress. Plant Signal. Behav..

[105] Lin Y., Yang L., Paul M., Zu Y., Tang Z. (2013). Ethylene promotes germination of *Arabidopsis* seed under salinity by decreasing reactive oxygen species: Evidence for the involvement of nitric oxide simulated by sodium nitroprusside. Plant Physiol. Biochem..

[106] Zhang S., Hu J., Zhang Y., Xie X.J., Knapp A. (2007). Seed priming with brassinolide improves lucerne (*Medicago sativa* L.) seed germination and seedling growth in relation to physiological changes under salinity stress. Aust. J. Agric. Res..

[107] Zhu T., Deng X.G., Tan W.R., Zhou X., Luo S.S., Han X.Y., Zhang D.W., Lin H.H. (2016). Nitric oxide is involved in brassinosteroid-induced alternative respiratory pathway in *Nicotiana benthamiana* seedlings’ response to salt stress. Physiol. Plant..

[108] Khan M.I.R., Asgher M., Khan N.A. (2014). Alleviation of salt-induced photosynthesis and growth inhibition by salicylic acid involves glycinebetaine and ethylene in mungbean (*Vigna radiata* L.). Plant Physiol. Biochem..

[109] Mostofa M.G., Fujita M., Lam-Son Phan T. (2015). Nitric oxide mediates hydrogen peroxide- and salicylic acid-induced salt tolerance in rice (*Oryza sativa* L.) seedlings. Plant Growth Regul..

[110] Liu S., Dong Y., Xu L., Kong J. (2014). Effects of foliar applications of nitric oxide and salicylic acid on salt-induced changes in photosynthesis and antioxidative metabolism of cotton seedlings. Plant Growth Regul..

[111] Dong Y., Wang Z., Zhang J., Liu S., He Z., He M. (2015). Interaction effects of nitric oxide and salicylic acid in alleviating salt stress of *Gossypium hirsutum* L.. J. Soil Sci. Plant Nutr..

[112] Hu X., Li W., Chen Q., Yang Y. (2009). Early signal transduction linking the synthesis of jasmonic acid in plant. Plant Signal. Behav..

[113] Yastreb T.O., Kolupaev Y.E., Karpets Y.V., Dmitriev A.P. (2017). Effect of nitric oxide donor on salt resistance of *Arabidopsis jin1* mutants and wild-type plants. Russ. J. Plant Physiol..

[114] Wang P., Dua Y., Hou Y., Zhao Y., Hsu C., Yuan F., Zhu X., Tao W., Song C.P., Zhu J.K. (2015). Nitric oxide negatively regulates abscisic acid signaling in guard cells by *S*-nitrosylation of OST1. Proc. Natl. Acad. Sci. USA.

[115] Albertos P., Romero-Puertas M.C., Tatematsu K., Mateos I., Sanchez-Vicente I., Nambara E., Lorenzo O. (2015). *S*-nitrosylation triggers ABI5 degradation to promote seed germination and seedling growth. Nat. Commun..

[116] Klein A., Keyster M., Ludidi N. (2015). Response of soybean nodules to exogenously applied caffeic acid during NaCl-induced salinity. S. Afr. J. Bot..

[117] Tanou G., Job C., Rajjou L., Arc E., Belghazi M., Diamantidis G., Molassiotis A., Job D. (2009). Proteomics reveals the overlapping roles of hydrogen peroxide and nitric oxide in the acclimation of citrus plants to salinity. Plant J..

[118] Qi Q., Dong Y., Liang Y., Li K., Xu H., Sun X. (2020). Overexpression of *SlMDHAR* in transgenic tobacco increased salt stress tolerance involving *S*-nitrosylation regulation. Plant Sci..

[119] Jain P., von Toerne C., Lindermayr C., Bhatla S.C. (2018). *S*-nitrosylation/denitrosylation as a regulatory mechanism of salt stress sensing in sunflower seedlings. Physiol. Plant..

[120] Wang C., Wei L., Zhang J., Hu D., Gao R., Liu Y., Feng L., Gong W., Liao W. (2022). Nitric oxide enhances salt tolerance in tomato seedlings by regulating endogenous *S*-nitrosylation levels. J. Plant Growth Regul..

[121] Camejo D., del Carmen Romero-Puertas M., Rodriguez-Serrano M., Maria Sandalio L., Jose Lazaro J., Jimenez A., Sevilla F. (2013). Salinity-induced changes in *S*-nitrosylation of pea mitochondrial proteins. J. Proteom..

[122] Ziogas V., Tanou G., Filippou P., Diamantidis G., Vasilakakis M., Fotopoulos V., Molassiotis A. (2013). Nitrosative responses in citrus plants exposed to six abiotic stress conditions. Plant Physiol. Biochem..

